# Dorland’s American Medical Dictionaries

**Published:** 1910-03-15

**Authors:** 


DORLAND'S AMERICAN MEDICAL DICTIONARIES. The well known Publishing House of W. B. Saunders & Co. of Philadelphia has just issued the Fifth Edition of Dorland's Illustrated Medical Dictionary and the Sixth Edition of the Pocket Medical Dictionary by the same author. These dictionaries are so well known and highly esteemed that it seems unnecessary to call attention to their exclusive merits. This new edition is a complete revision and contains more than 2000 words not in the first edition. It is the latest and best up-to-date authoratative work of its kind. The author has succeeded in making his definitions so terse and yet ample as to keep the book within reasonable size for convenient handling and use. At the same time it is generously supplied with plates of illustrations, many in colors, illustrating ideas for which great space would be required to make intelligent descriptions. The book is well printed and bound in red limp leather and is designed for constant usage.

The Pocket Edition is similarly made and is a marvel of completeness and compactness. It contains a concise definition of all the terms of the larger work but not so many of the valuable tables and none of its valuable illustrations. Of course it can not give word derivation or extended definition, but for quick reference it has its place. Every student especially should possess a good up-to-date dictionary and the small cost of such a work should be no excuse for not having such as will give the most information. We should urge the purchase of the illustrated edition as it answers all questions so much more completely that its comparative cost is not worth considering. Just think of having a book always at hand for an outlay of less than five dollars which will instantly give you a complete answer as to spelling, pronunciation, derivation and meaning of any word you will meet in all your medical reading or experience. Dentists should throw away that old Medical Dictionary that was a back number twenty years ago, and take some comfort in the possession of such a modern aid as is now readily available. One of the reasons why many dentists don't read scientific articles in their journals is because they don't understand them. With the aid of a first class dictionary all this literature would become interesting and a mental development would result that will be of immense practical value.

